# Excitatory and inhibitory synaptic mechanisms at the first stage of integration in the electroreception system of the shark

**DOI:** 10.3389/fncel.2014.00072

**Published:** 2014-03-06

**Authors:** Naama Rotem, Emanuel Sestieri, Jorn Hounsgaard, Yosef Yarom

**Affiliations:** ^1^The Otto Loewi Center, The Inter University InstituteEilat, Israel; ^2^Department of Neurobiology, The Edmond & Liliy Safra Centre for Brain Sciences, Life Science Institute, Hebrew UniversityJerusalem, Israel; ^3^Department of Neuroscience and Pharmacology, The PANUM Institute, University of CopenhagenCopenhagen, Denmark

**Keywords:** dorsal octavolateral nucleus, electroreception, shark, afferent, parallel fibers, feed forward inhibition, cancellation mechanism

## Abstract

High impulse rate in afferent nerves is a common feature in many sensory systems that serve to accommodate a wide dynamic range. However, the first stage of integration should be endowed with specific properties that enable efficient handling of the incoming information. In elasmobranches, the afferent nerve originating from the ampullae of Lorenzini targets specific neurons located at the Dorsal Octavolateral Nucleus (DON), the first stage of integration in the electroreception system. Using intracellular recordings in an isolated brainstem preparation from the shark we analyze the properties of this afferent pathway. We found that stimulating the afferent nerve activates a mixture of excitatory and inhibitory synapses mediated by AMPA-like and GABA_A_ receptors, respectively. The excitatory synapses that are extremely efficient in activating the postsynaptic neurons display unusual voltage dependence, enabling them to operate as a current source. The inhibitory input is powerful enough to completely eliminate the excitatory action of the afferent nerve but is ineffective regarding other excitatory inputs. These observations can be explained by the location and efficiency of the synapses. We conclude that the afferent nerve provides powerful and reliable excitatory input as well as a feed-forward inhibitory input, which is partially presynaptic in origin. These results question the cellular location within the DON where cancelation of expected incoming signals occurs.

## Introduction

A wide range of exteroceptive sensory modalities are mediated by hair cells. The ongoing transmission at the ribbon synapse and the ensuing high spike rates in the afferent axons are metabolically costly but presumably serve to secure sensitivity while accommodating a wide, bidirectional dynamic range (Clusin and Bennett, 1979; Wen et al., 2009; Ospeck, 2012). The persistently high afferent impulse rate poses special requirements on the recipient synaptic machinery such as high transmission fidelity as well as efficient integrative capabilities.

In the shark electroreception sense, afferent nerves originate at hair cells located in the sensory organ known as the ampullae of Lorenzini. The high impulse rate of this nerve encodes the level of external electric field (Tricas and New, 1998). Unlike other exteroceptive sensory modalities, the ampullary afferents project exclusively to the Dorsal Octavolateral Nucleus (DON) to excite the principal ascending efferent neurons (AENs) as well as interneurons that provide the principal neurons with feed-forward inhibition (Duman, 1997; Oertel and Young, 2004; Biesdorf et al., 2008). Accumulating evidence suggests that the internal circuitry within the DON provides the mechanism that can discriminate between external and self-generated electric fields. To date, most of the studies are based on unit recordings from intact system that although accurately represent the responses of the neurons, are inadequate to examine synaptic mechanisms that are an essential step toward understanding how AENs integrate the electrosensory inputs.

In the present study we use intracellular recordings from AENs in the isolated brainstem preparation of the shark (Rotem et al., 2007) to analyze the synaptic inputs evoked by activation of the electrosensory nerve. Our results show that each AEN is contacted by a small number of afferents each of which establishes a highly efficient excitatory connection that operates as a current source. This excitatory input is followed by a powerful inhibitory input that markedly reduces the afferent excitation.

## Materials and methods

### The animal

120 Adult female and male *Iago omanensis* sharks were caught in the Gulf of Eilat, from a depth range of 400–800 m. Sharks, 30–60 cm in length, were collected at night, using a red light source to prevent eye damage, and kept at 20°C in a dark seawater pool with fresh seawater circulation rate of 20% in 24 h.

### The preparation

The isolated brain stem preparation has been described in a previous publication (Rotem et al., 2007). Briefly, the brain stem and the afferent nerve were isolated and incubated in the experimental chamber with continuously superfusion of aerated, 20°C Ringer solution. The shark Ringer solution (modified from Hentschel et al., 2003), contained (in mM) 280 NaCl, 6 KCl, 5 CaCl2-2H2O, 3 MgCL2-6H2O, 0.5 Na2SO4, 1 NaH2PO4-12H2O, 8 NaHCO3, 350 urea, 72 trimethylamine N-oxide dehydrate (TMAO, Sigma, Rehovot, Israel), 5 glucose, 0.75 polyvinylpyrrolidone (PVP-40T, Sigma).

### Recordings and stimulation

Sharp glass pipettes filled with 2 M potassium acetate at 30–60 MΩ were used for Intracellular recording. An axoclamp 2A amplifier, in current clamp bridge mode configuration, was used for recordings. Data acquisition board (PCI-MIO-16XE-10, National Instruments, Austin, TX, USA), controlled by software written in LabView (National Instruments), was used to sample the data at a rate of 10,000 kHz and stored for offline analysis. Bipolar stimulating electrodes were placed on the afferent nerve stump and on the surface of the DON for activation of the parallel fibers.

### Pharmacology

Bicuculline, a GABA_A_ receptor blocker, was applied at a final concentration of 50–100 μM. Gabazine (SR-95531, Sigma), a reversible GABA_A_ receptor blocker, was applied at a final concentration of 300 nM. CNQX, a glutamatergic AMPA receptor blocker, was applied at a final concentration of 25 μM. Bicuculline, gabazine, and CNQX were added to the external Ringer solution and the recording started after approximately 30 min. In some experiments the recording electrode was filled with QX- 314, an intracellular Na+ channel blocker, (100 mM dissolved in 2 M KAc) or QX-314 and CsCl, an intracellular K+ channel blocker (1 M CsCl, 100 mM QX-314 and 1 M KAc). Positive current pulses of 0.5–1.2 nA in amplitude, 50–100 ms in duration repeated at 1 Hz were used to deliver the drugs to the recorded cell.

### Analysis

The amplitudes of both the action potentials and the synaptic potentials were measured from the resting potential to the peak of the response. Duration was measured at half amplitude. The rise time of the synaptic potentials was measured from 10 to 90% of the amplitude. The reversal potential of the inhibition was measured by the voltage of the membrane potential at which the IPSP reversed polarity. The synaptic delay was measured as the time between the end of stimulus and beginning of the response. Voltage threshold was measured from the plot of the voltage derivative as a function of membrane voltage (dV/dt as a function of V). Voltage dependence of the synaptic potentials was calculated by measuring the synaptic potential amplitude at different membrane potentials (by applying different current steps) and normalizing the amplitude to the synaptic potential at rest membrane potential. The slop of the voltage dependent relation was calculated for each cell separately by calculating the slope of the curve. The average voltage dependent relation (Figure 6C, red line) was calculated by averaging all slopes from all the cells in this experiment.

Sub-threshold synaptic potentials were occasionally averaged five times. In each experiment the calculated values are giving as percentage or as average ± SD and the number of *N*. In the experiment that tested the relation between depth of the recording sites, rise time and duration we calculated the correlation coefficient for linear regression using excel software.

## Results

### Primary afferent input to ascending efferent neurons in the DON

In this study we analyzed intracellular recordings from 238 AENs in 60 sharks. Cells were identified by their response to afferent and parallel fiber input as well as input resistance in the range of 10–30 MΩ. In our previous work (Rotem et al., 2007) we labeled neurons with neurobiotin and found that all of them where large neurons with apical dendrite(s) ascending toward the molecular layer and with a number of basal dendrites. These cells had resting potentials of at least −50 mV and action potential amplitudes higher than 50 mV. The average resting potential and the average action potential amplitudes was −69.2 ± 10.1 mV and 64.2 ± 10.1 mV (*n* = 80), respectively.

**Figure 1:**
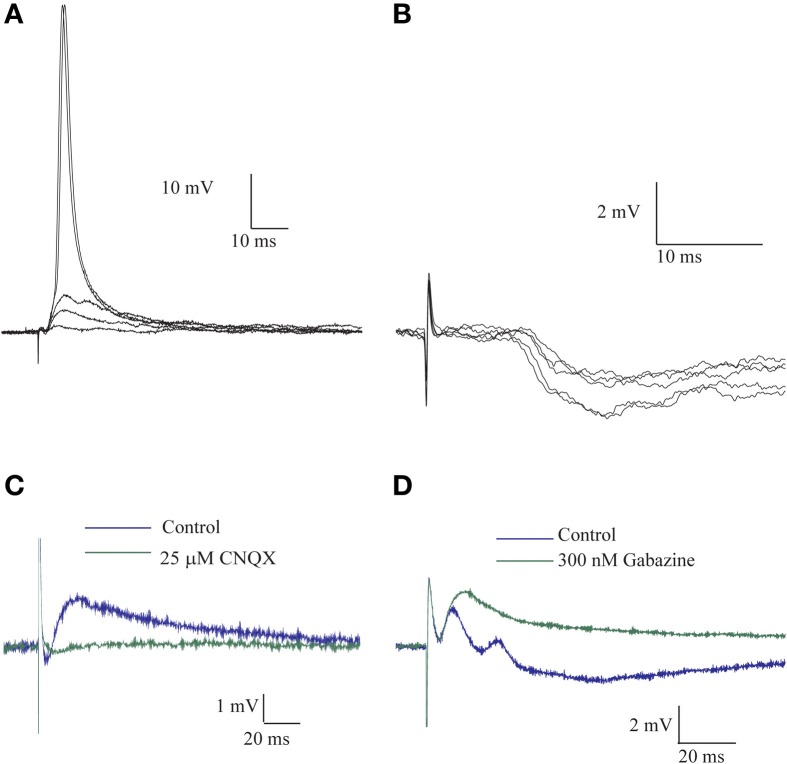
**The afferent input to AENs evokes a mixture of excitatory and inhibitory synaptic responses**. All traces are Intracellular recording from an AEN. **(A)** The excitatory response of AEN to stimuli delivered to the afferent nerve at gradual increase in stimulus intensity. **(B)** As in **(A)** for a case of Inhibitory responses. **(C)** Application of CNQX completely blocked the response to afferent nerve stimulation. **(D)** In a different preparation, application of gabazine blocked the inhibitory response and revealed the underlying excitatory response.

Stimulating the afferent nerve usually evoked biphasic responses where a depolarizing synaptic potential was followed by a hyperpolarizing response. The latter was regularly revealed by shifting the membrane potential to depolarized levels. The amplitude of the depolarizing response increased with stimulus intensity and readily reached threshold for the action potential (Figure 1A). This depolarizing response, in which two or three components were often distinguishable (Figure 1D), had an average delay of 2.4 ± 1.2 ms (*n* = 8), an average rise time of 4.0 ± 2.2 ms (*n* = 67) and an average duration of 31.5 ± 6.4 ms (*n* = 20). The depolarizing and the hyperpolarizing responses were blocked by CNQX, a specific AMPA antagonist (Figure 1C), while the specific GABA_A_R antagonist, gabazine, blocked the hyperpolarizing response, leaving a monophasic depolarizing response (Figure 1D). The effect of gabazine on the amplitude of the depolarizing responses was examined in 21 cells. No effect was observed in 40% of the cells, a decrease in amplitude was observed in another 40% of cells and increase in the remaining 20%.

Monophasic hyperpolarizing responses (Figure 1B), which were only rarely encountered (3 cells out of 238), had an average delay of 7.8 ± 1.5 ms, an average rise time of 14.7 ± 6.2 and an average duration of 156.6 ± 106.9 ms (*n* = 3).

These results indicate that afferent input to AENs evokes a mixture of excitatory (EPSP) and inhibitory (IPSP) synaptic responses and thus indicates the involvement of inhibitory interneurons. Furthermore, the short delay of the inhibitory synaptic response is in line with involvement of feed-forward inhibition.

### Conductance and voltage sensitivity of the afferent synaptic response

**Figure 2:**
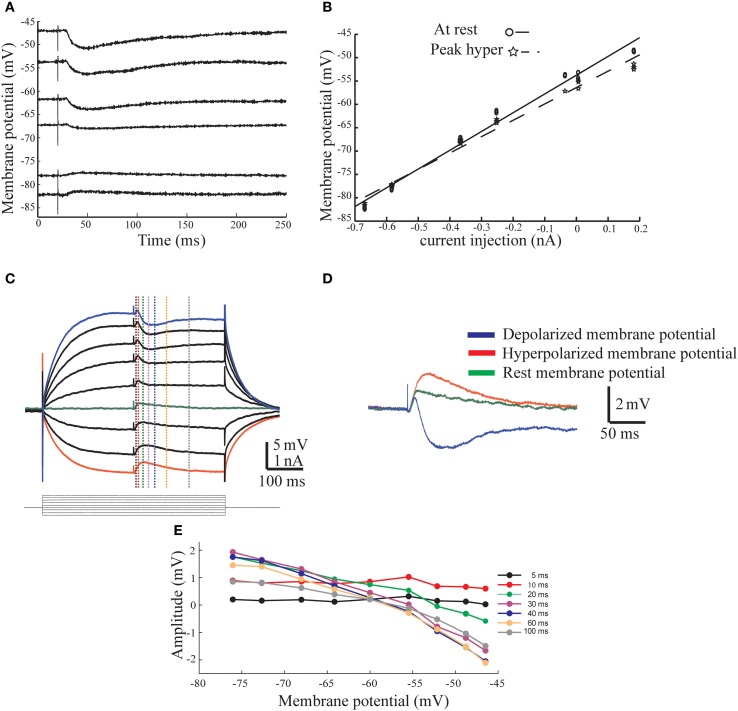
**The conductance base of the response evoked by afferent nerve stimulation. (A)** Inhibitory response evoked by afferent nerve stimulation from different levels of membrane potential. Note a clear reversal of the response. **(B)** The current voltage relationship measured at the peak of the response (dashed line) and before the stimulus onset (continues line). **(C)** Excitatory response evoked by afferent nerve stimulation from different levels of membrane potential Note the negative/inhibitory phase in depolarized membrane potentials. **(D)** Three superimposed traces (from **C**) show the responses at the depolarized (blue), hyperpolarized (red), and at rest (green) membrane potential. Note the temporal relationship between the three responses. **(E)** The amplitude of the response measured at six different time points as function of membrane potential. The time of measurements is indicated in **(C)** with dashed lines in corresponding color.

**Figure 3:**
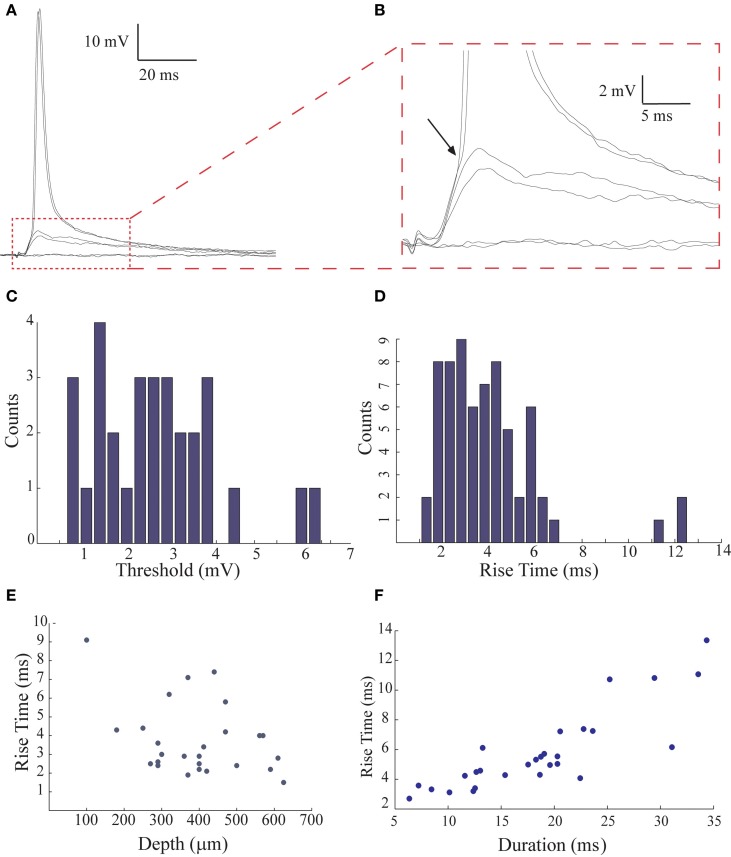
**The low threshold for spike initiation and the fast rise time of the post synaptic responses evoked by afferent nerve stimulation. (A,B)** Sub- and suprathreshold responses evoked by afferent nerve stimulation displayed at different scales. Arrow in **(B)** indicates the deflection points in the voltage trace that correspond to the threshold. **(C)** The distribution of the threshold level for spike generation measured from 30 AENs. **(D)** The distribution of the rise time of the response (*n* = 67). **(E)** The rise time of the responses is negatively correlated with the depth of the recording (*n* = 26; the correlation coefficient for linear regression was 0.12). **(F)** The rise time is positively correlated with its duration (*n* = 28; the correlation coefficient for linear regression was 0.72).

To further characterize the afferent input to AENs we calculated the conductance and the reversal potential for the synaptic responses. To this end we measured the amplitude of the synaptic potentials evoked by stimulating the afferent nerve at different membrane potentials. As illustrated in Figures 2A,B, the IPSP behaved as expected from a simple conductance change process with a reversal potential of −73 mV and a conductance of 0.28 μS (Figure 2B, dashed line). On average, the IPSP reversal potential was −64 ± 7.5 mV and the conductance was 0.31 ± 0.18 μS (*n* = 3). The excitatory synaptic potential (EPSP) (Figures 2C,D) displayed more complex behavior, manifested by a delayed hyperpolarizing phase that appeared with depolarizing current injections. The three superimposed traces in Figure 2D show the responses at the most depolarized (blue) and hyperpolarized (red) membrane potentials as well as without current injection (green). The temporal relation between the different phases supports the conclusion that the afferent nerve stimuli evoke short-latency excitatory synaptic responses followed by synaptic inhibition with a slightly longer latency. The amplitude of the synaptic potential was measured at different times along the compound response (dotted line in Figure 2C) and plotted as a function of the membrane potential (Figure 2E). Whereas the response during the first 10 ms showed voltage independence (Figure 2E, black and red curves; see also Figures 3, 6), a reduction in amplitude with membrane depolarization is evident at all-time points after 20 ms and the responses measured after 40 ms all reverse at −60 mV. The average reversal potential of the inhibitory component calculated from 11 AEN's was −44.1 ± 37.9 mV and the conductance measured at the peak of the hyperpolarized phase was 0.39 ± 0.29 μS. This lower reversal potential as well as the high degree of variability is probably due to variable contribution of excitatory conductance at the time of measurement.

We conclude that the primary afferent input to the DON directly excite the AENs and provide them with short latency inhibition. In the following sections, we analyze the properties of the excitatory and inhibitory innervation.

### The excitatory afferent input to the AEN

A characteristic feature of the primary sensory transmission is the low threshold for afferent activation. This is demonstrated in Figure 3. Gradual increase in stimulus intensity evokes an all-or-none synaptic potential that occasionally reaches spike threshold (Figure 3A; see also Figure 1A). The enlarged trace in Figure 3B shows that the threshold is readily defined by a deflection in the depolarizing trajectory (arrow, see Methods). The average threshold in 30 neurons that had resting potentials of −69 ± 7 mV was −67 ± 8 mV. Accordingly, the relative threshold was 2.64 ± 1.35 mV (*n* = 30; Figure 3C). In previous work (Rotem et al., 2007) we suggested that the low threshold could be due to close proximity of the location of the afferent synapse (presumably on the basal dendrites) to the spike initiation zone of the axon. In the present work we noticed that the rise time of the synaptic potentials (calculated as 10–90% of the peak amplitude), was relatively widely distributed (average 3.95 ± 2.23 ms, range 1–7 ms, *n* = 67; Figure 3D). This could reflect the range of distances between the location of afferent synapses and the recording sites. Assuming that most of the intracellular recordings in the molecular layer are from apical dendrites of AENs (Rotem et al., 2007), a deeper recording sites should be closer to the basal dendritic and thus to the origin of the synaptic potentials. Indeed, the depth of the recording and the synaptic rise time were inversely correlated (*R*^2^ = 0.13; *p* < 0.06; Figure 3E; *n* = 26). This is further supported by the correlation between the synaptic potential rise time and duration at half amplitude (*R*^2^ = 0.72; *p* < 0.0001; Figure 3F; *n* = 28; Rall et al., 1967).

**Figure 4:**
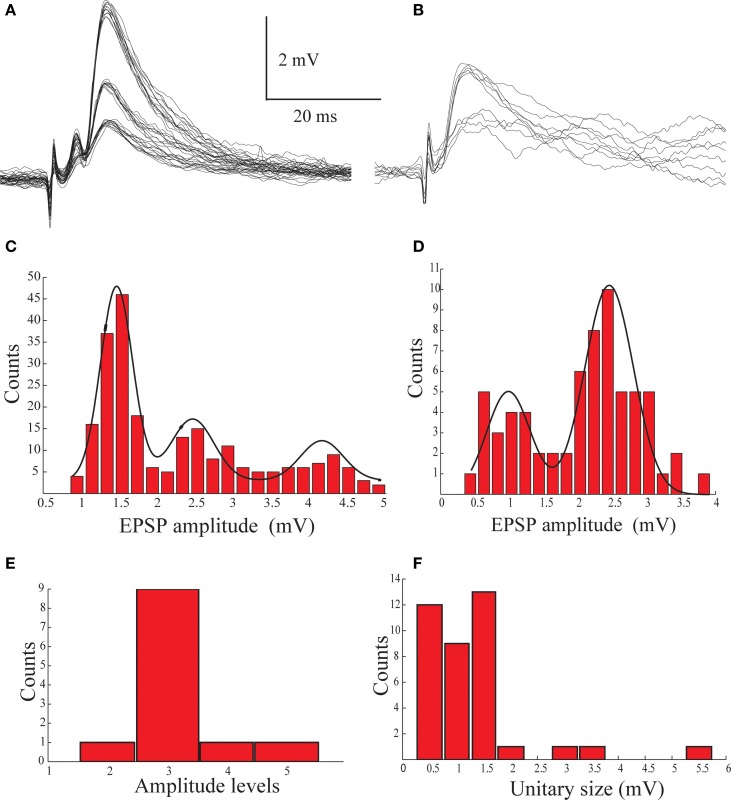
**The multimodal distribution of the synaptic potentials evoked by afferent nerve stimulation. (A,B)** An example of synaptic potentials recorded from two AENs following gradual increase in stimulation intensity of the afferent nerve. **(C,D)** Histograms of the amplitude distribution of the traces shown in **(A,B)** plotted as function of stimulus intensity and fitted by Gaussians (black lines), showing bi- and triple-modal distribution. **(E)** The distribution of the number of peaks in the amplitude histogram of 12 AENs. **(F)** The distribution of the inter peaks intervals measured from the amplitude histogram of 12 AENs.

We conclude that the electro-sensory afferents terminate on basal dendrites of AENs at the ventral side of the nucleus, close to the spike initiation zone and that these synapses are highly efficient in activating the neurons. We therefore expect low convergence of the afferent fibers on AENs. In order to examine this point we measured the dynamic range of the excitatory afferent input while blocking action potentials in the recorded cell by intracellular injection of QX-314. Figures 4A,B display examples of postsynaptic potentials (PSPs) recorded from two AENs following a gradual increase in stimulus intensity. In both AENs the response amplitudes clustered into distinct groups. It should be noted that even at a given stimulus intensity the amplitude of the response varied between several discrete values but the probability to respond with higher amplitude increased with stimulus intensity. The amplitude distributions of the responses, calculated by measuring the amplitude of the different traces from the two AENs in Figures 4A,B revealed clustered groups of PSP amplitudes (Figures 4C,D). These distributions were fitted by 2 and 3 Gaussians, suggesting bi- and tri-modal distributions with similar inter-peak-intervals of about 1.2–2 mV, define as the amplitude of a unitary event. The population statistic of the unitary events amplitude from 12 AEN's is shown in Figures 4E,F. The number of unitary events ranged from 2 to 5, where three events were most commonly observed (Figure 4E). The average size of the unitary event was 1.36 ± 0.99 mV (*n* = 12) (Figure 4F), which is similar to the amplitude of the minimal response to afferent nerve stimulation (Figures 4A,B). Taken together, these results support the possibility that the unitary events represent activation of individual afferent fibers each of which upon activation releases a similar amount of neurotransmitter. We conclude that each AEN is targeted by highly efficient synapses formed by a small number of afferent fibers. Such an arrangement suggests high spatial resolution of electroreception (see discussion). Such amplitude grouping most likely to occurs if individual AENs are innervated by a small number of afferent fibers, each of which evokes an all-or-none response.

### The biophysical properties of the excitatory afferent input to the AEN

**Figure 5:**
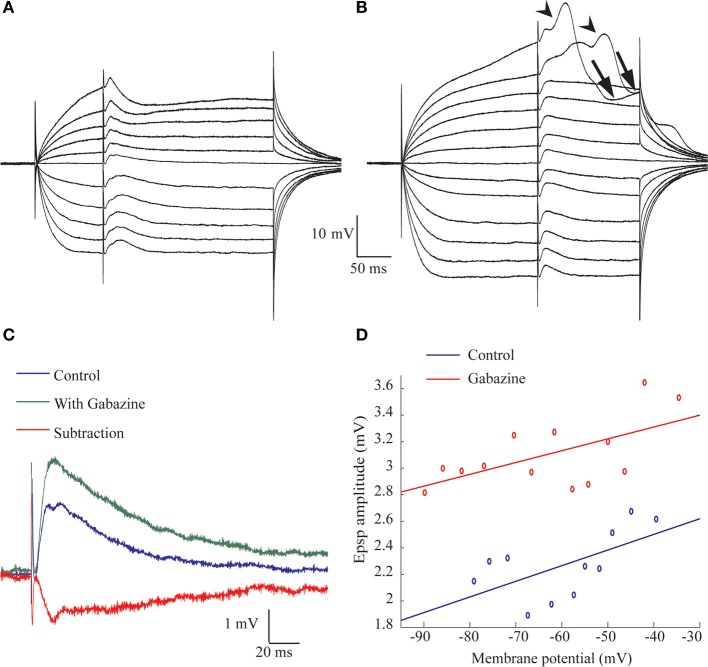
**Blocking GABAergic transmission reveal the excitatory response evoked by afferent nerve stimulation. (A,B)** The voltage dependent of the responses to afferent nerve stimulation before **(A)** and after **(B)** application of 300 nM gabazine. **(C)** Superimposed traces taken from **(A,B)** at rest membrane potential before (blue trace) and after (green trace) application of gabazine. The difference is shown in red. Note the early onset of the inhibitory response and the similarity in time course of the excitatory and inhibitory response. **(D)** The amplitude of the response as a function of membrane potential calculated from (**A**, blue) and (**B**, red). Note the increase in amplitude with membrane depolarization.

**Figure 6:**
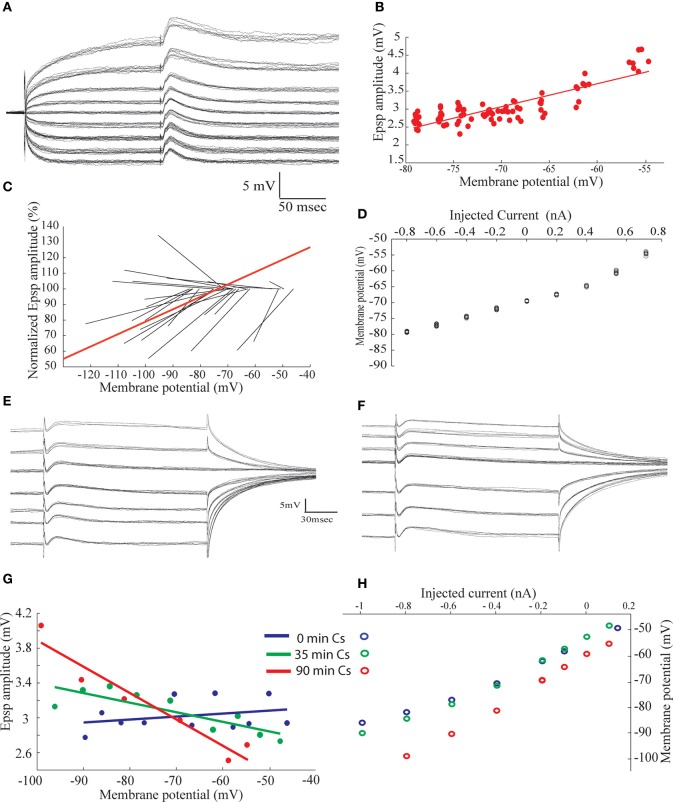
**The amplitude of the excitatory synaptic potentials evoked by afferent nerve stimulation increases with membrane depolarization. (A)** Synaptic potentials response evoked by afferent nerve stimulation recorded at different membrane potentials. **(B)** The amplitude of the synaptic potentials plotted as a function of the membrane potential. **(C)** Normalized regression lines of the voltage dependent of the synaptic potentials measured in 24 neurons. Red line is the calculated average relationship. **(D)** The voltage current relationship of the neuron shown in **(A)**. **(E,F)** Synaptic potentials evoked by afferent nerve stimulation superimposed on different levels of membrane voltage, before **(E)** and 90 min after **(F)** penetrating the cell with Cs filled electrode. **(G)** The amplitude of the synaptic potential as a function of the membrane potential at the beginning of the recording (blue) after 35 min (green) and after 90 min (red). **(H)** The current voltage relationship at the beginning of the recording (blue) after 35 min (green) and after 90 min (red).

The amplitude of the excitatory component of the synaptic response was surprisingly insensitive to membrane potential (Figure 2E). To investigate the underlying mechanisms we first measured the response before and after blocking GABA_A_ receptors in the presence of QX-314 in the recording pipette. As shown in Figures 5A,B, the delayed hyperpolarizing phase that appear when the membrane potential is depolarized beyond −45 mV is blocked by gabazine (Figure 5B) whereas the initial depolarizing phase increases (Figure 5C, green trace). Subtracting the responses with and without gabazine revealed that the hyperpolarizing responses had a time course similar to the depolarizing response (Figure 5C, red trace). Similar results were obtained from eight AEN's. As a result of the blockade of the inhibition, the excitatory component reaches threshold at depolarized membrane potentials, and occasionally triggers a slow, calcium-dependent regenerative responses (arrows heads in Figure 5B) followed by after hyperpolarization (arrows in Figure 5B). Unexpectedly, the excitatory response increased in amplitude with depolarization (Figure 5D) both before (Figure 5D, blue) and after (Figure 5D, red) gabazine application. This surprising observation prompted a thorough examination of the biophysical properties of the excitatory input with gabazine (0.3 μM) or bicuculline (50 μM) added to the extracellular medium in all experiments. The afferent nerve was stimulated during stepwise depolarizing and hyperpolarizing shifts in the membrane potential and the resulting EPSP amplitude was measured and plotted as a function of the membrane potential(Figure 6B), revealing a linear positive slope of 0.064. This relationship was studied in 24 neurons shown in Figure 6C where the regression lines of the relationship between the normalized EPSP amplitude and the membrane potential are plotted (see methods). In all but four neurons the relation between membrane potential and the amplitude of the evoked excitatory response was positive with an average slope of 0.019 ± 0.033.

There are two plausible explanations for such relationships. Either the electrical properties of the membrane are non-linear or the ionic channels that are activated by the neurotransmitter display unconventional properties (such as a decrease of the potassium conductance simultaneous with increase of sodium conductance; Brown et al., 1971; Adams et al., 1980). As shown in Figure 6D, the I-V relationship is nonlinear, with the membrane resistance (as measured from the slope of the I-V curve) changes from 10 to 30 MΩ when the membrane is depolarized. Furthermore, the EPSP duration is longer at depolarized membrane potentials. Although nonlinearity of the membrane resistance can account for the increase in EPSP duration, it cannot explain the increase in amplitude with depolarization (see Discussion).

In order to further examine the peculiar behavior of this excitatory synapse and elucidate the role of membrane nonlinearity we added cesium to the recording pipette. Cesium ions are potent blocker of potassium channels and tend to linearize the I-V relation of neuronal membranes when diffusing out of the recording pipette to the cytosol. The relation of EPSP amplitude to membrane potential was investigated at two different times after penetration with a Cs-filled electrode as shown in Figures 6E,F. Immediately after penetration the synaptic potential behaves similarly to what was shown in Figure 6A, that is, the amplitude and duration of the synaptic potential increased with depolarization (Figure 6G, blue curve; positive slope of 0.0041). Ninety minutes after penetration, an increase in input resistance is evident (Figure 6H), particularly at more negative potentials, and the relation between amplitude and membrane potential attain a linear negative slope (−0.03) (Figure 6G, red). It should be noted that negative slope was already reached after 35 min of recording (Figure 6G, green), demonstrating that minimal change in input resistance (Figure 6H, green) is sufficient to reverse the relationships. It thus can be argued that the effect of Cs is solely mediated by linearization of the membrane electrical behavior (see Discussion)

### Feed forward inhibition of AENs

**Figure 7:**
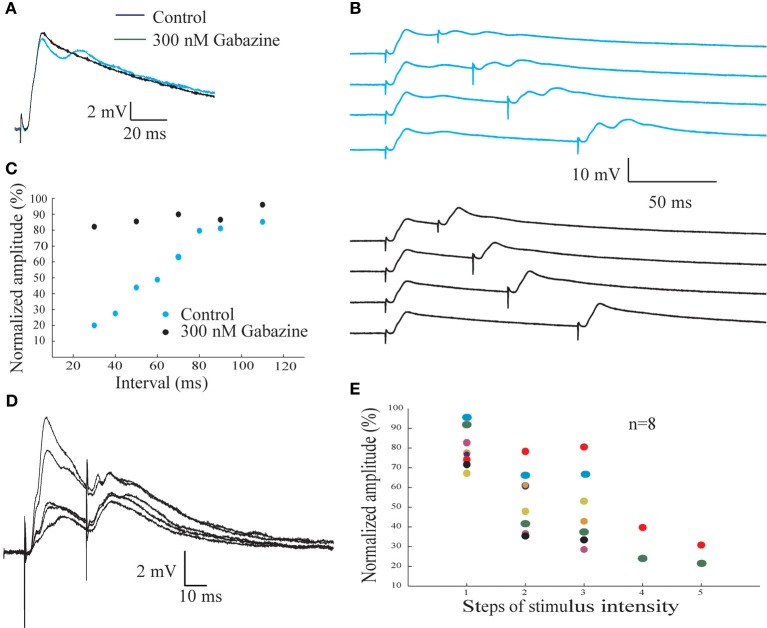
**The inhibitory input to the AEN revealed by afferent nerve paired pulse stimulating protocol. (A)** Synaptic potentials response evoked by afferent nerve paired pulse stimulation before (blue trace) and after (black trace) gabazine application. **(B)** Afferent nerve paired pulse stimulation protocol at 30, 50, 70, and 110 m intervals before (upper pane l) and after (lower panel) gabazine application. **(C)** The normalized amplitude of the second synaptic response as a function of the interval between the stimuli before (blue circles) and after (green circles) the addition of gabazine. **(D)** The responses of the AENs to afferent nerve pair pulse stimulation at various intensities, delivered at an interval of 30 ms. The increase in the response for the first stimulus is accompanied by a larger decrease of the response to the second stimulus. **(E)** The normalized amplitude of the second response as a function of the intensity of the first stimulus. Each color represent individual cell.

Previously we have demonstrated using extracellular recordings that paired-pulse stimulation protocol reveals a powerful inhibition mediated by GABA_A_ receptors (Rotem et al., 2007). Here we examine this inhibition further using intracellular recordings (Figure 7). Paired-pulse stimulation was delivered to the afferent nerve at different inter-stimulus intervals (Figure 7B). A substantial reduction in amplitude of the second response, which depends on the inter-stimulus interval, is evident (upper blue traces in Figure 7B). The peak amplitude of the response to the second stimulus was measured, normalized by the amplitude of the response to the first stimulus and plotted as a function of the inter-stimulus interval (Figure 7C, blue symbols). The reduction in the amplitude of the second response increases as the inter-stimulus interval decreases, reaching a maximum of 80% reduction at 30 ms interval. This protocol was repeated in the presence gabazine. Although the amplitude of the response was only slightly affected (compare blue and black traces in Figure 7A), the inhibition revealed in paired pulse protocol was completely blocked (black traces in Figure 7B and black symbols in Figure 7C).

The observation that powerful inhibition revealed by pair pulse protocol is associated with rather small postsynaptic hyperpolarization suggests that either the inhibition is pre-synaptic in origin or spatially isolated post-synaptically. To further examine this point we performed two sets of experiments. First, we studied the effect of stimulus intensity on the inhibition. Second, we examined the effect of the afferent evoked inhibition on parallel fiber input.

Using paired-pulse interval of 30 ms and gradual increasing the afferent nerve stimulation intensity we found that the paired-pulse depression is not linearly related to the stimulus intensity (Figures 7D,E). A step like increase in inhibition occurs at a certain change in stimulus intensity. At low stimulus intensity the amplitude of the response to the second stimulus is almost (79.6 ± 9.8%; *n* = 8) as large as the amplitude of the first response. At high stimulus intensity, the amplitude reached 26 ± 6.7% (*n* = 2) of the control level, whereas at medium stimulus intensity an average reduction of 48.8 ± 18% (*n* = 7), was measured.

Two important concepts seem to emerge from these observations. First, since inhibition is negligible at low stimulus intensities, one must conclude that excitation of AENs can be elicited without being followed by inhibition. Second, upon increasing the stimulus intensity, the inhibitory effect seems to saturate before excitation, suggesting that only a small number of fibers are responsible for the inhibition of each AEN. This is also supported by the high variability in inhibition at intermediate stimulation intensities (see Discussion).

**Figure 8:**
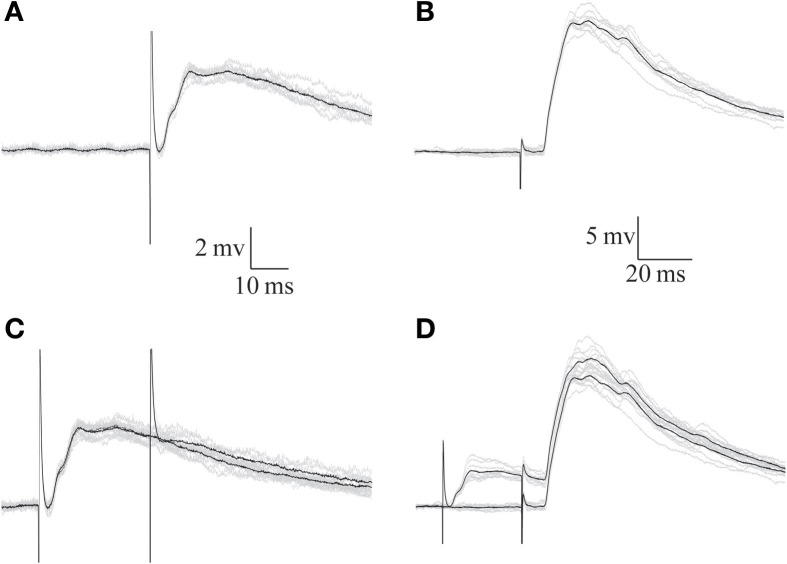
**The response evoked by parallel fibers stimulation is insensitive to the inhibition evoked by afferent nerve stimulation. (A,B)** Synaptic potentials evoked by afferent nerve **(A)** and parallel fibers **(B)** stimulation. **(C,D)** Preceding the responses shown in **(A,B)** by afferent nerve stimulation eliminates the response to the same stimulus **(C)** but does not affect the response to parallel fibers stimulation **(D)** Black traces show the averages of the 10 traces (gray) in each panel.

To examine the inhibitory effect of afferent nerve stimulation on parallel fiber-evoked responses, stimulating electrodes were placed both on the afferent nerve and on the surface of the DON to activate the parallel fibers system. Stimulation at either location evoked depolarizing postsynaptic responses (Figures 8A,B). However, the afferent nerve-evoked response was completely eliminated when preceded by another afferent nerve stimulus (Figure 8C) while the parallel fiber-evoked response was unaffected (Figure 8D). Results similar to those shown in Figure 8 were obtained in 7 experiments in which the reduction in parallel fibers response by a preceding afferent stimulus was only 7.8 ± 10.6% while the afferent response was reduced by 88.6 ± 10.9 % (*n* = 7). Thus, we conclude that the inhibition evoked by afferent fibers is highly localized in AENs.

## Discussion

In this study we characterized, for the first time the synaptic potentials evoked in AENs by stimulating the afferent electrosensory nerve. Beyond the immediate relevance to electroreception in elasmobranches, our study adds to the general understanding of early processing of hair cell sensation in the central nervous system. In the discussion we focus on the four main findings: (a) the low afferent convergence on AENs, (b) the electrotonic separation of the afferent input from the parallel fibers input in AENs, (c) the unusual voltage dependence of afferent EPSPs in AENs and (d) the properties and mechanisms of the feed-forward inhibition.

### Compartmentalization and low convergence of afferent excitation in AENs

Previous work suggested that the afferents nerve terminate strategically on AENs in the vicinity of the spike initiation zone (Rotem et al., 2007). We presently corroborate this possibility by analyzing the shape-indices of the synaptic potentials (Rall et al., 1967), demonstrating that the afferent nerve terminate at the basal dendrites or cell body of AENs close to the spike initiation zone (Figures 3E,F). Furthermore, by analyzing the distribution of the synaptic potentials amplitude, we demonstrated clustering into distinct, evenly distributed groups (Figure 4). Such distinct clustering can emerge either by activation of individual fibers or by the quantal nature of synaptic transmission. Since clustering also occurs when the stimulus intensity is increased, we conclude that it represents the variability in number of individual activated fibers. Thus, each fiber contributes about 1 mV to the overall excitatory postsynaptic response and that each AEN is innervated by 2–5 fibers. This low number of fibers suggests that the receptive field of a AEN comprises of only a very small number of ampullae. Each ampulla is the origin of 6–10 afferent nerve fibers (Bodznick and Schmidt, 1984; Fields and Ellisman, 1985) and *Iago omanensis*, the shark we investigate, has about 1400 ampullae (Fishelson and Baranes, 1998). This sums up to about 12,000 afferent nerve fibers. Based on previous work we estimate that the DON consists of about 2000 AENs (Paul and Roberts, 1977a). Thus, based on our present results and the anatomical information, we conclude that in this pathway the convergence and divergence is very low. Such an arrangement results in high spatial resolution of this sensory system, enabling detection of very small objects. Interestingly, the electroreceptive system in paddlefish provides the exquisite spatiotemporal perceptive resolution (Pothmann et al., 2012).

### The unusual voltage dependency of the synaptic potential

The amplitude of the EPSP increased upon membrane depolarization (Figures 5D, 6B). This unusual voltage dependency was inverted when Cs^+^, a non-specific intracellular blocker of K^+^ currents (Sierra et al., 2007; Weiger et al., 2007), was included in the electrode (Figure 6). This change was accompanied by an increase in input resistance and linearization of the current-voltage relationships. It is tempting to speculate that under control conditions intrinsic conductances modulate the amplitude of the EPSP in such a way that the increase in input resistance on depolarization increases the amplitude of the synaptic potential more than the expected decrease due to reduced driving force. Alternatively, the increased input resistance on depolarization will shorten the electrotonic length of the neuron, thereby closing the distance between the recoding site and the synaptic site. As a result, an increase in EPSP amplitude is expected. Again, if this increase is larger than the expected decrease, a positive relationship between EPSP amplitude and membrane voltage is bound to occur. However, if the membrane non-linearity is the only mechanism involved, one would expect Cs^+^ treatment to increase the amplitude of the EPSP over the entire range of membrane potentials and hence, it cannot account for the decrease in amplitude of the EPSP at depolarizing levels as shown in Figure 6G (at the range between −70 and −50 the EPSP amplitude after Cs treatment, green and red curves, is lower than that of the control, blue curve) There are two plausible explanations for this observation. First, the increased membrane resistance following Cs injection will compress the electrotonic structure of AENs. As a result, the voltage change at the synaptic site, which is induced by current injection at the recording site, will reach higher values, resulting in a decrease in the synaptic driving force and a reduction in the amplitude of the synaptic potential. Thus, at the site of recording a smaller synaptic potential will be recorded for the same membrane potential.

The second possibility is that an unusual synaptic conductance is involved, in which an increase in conductance for inward current is accompanied by a decrease in conductance for outward current. In such a synapse, which has been described *in Aplysia* neurons and was treated theoretically, the EPSP amplitude is independent of the membrane potential (Brown et al., 1971; Adams et al., 1980). If we assume that Cs^+^ blocks the decrease in outward current during the activation of the synapse, a reduction in EPSP amplitude is expected. Furthermore, following the blockade of synaptic decrease in outward current, the evoked EPSP is generated by only an increase in inward current and therefore the amplitude will decrease on depolarization. It is difficult to assess which of these two possibilities describe the mechanism of afferent transmission in AENs. However, the linear relation between amplitude and membrane potential requires an almost perfect compensation of the reduction in driving force by change in input resistance that is difficult to explain.

Regardless of the biophysical mechanism, our results indicate that the amplitude of the EPSP is almost independent of membrane potential. Therefore, these synaptic potentials can be treated as a current source, implying a linear integration of the input. Thus, the contribution of each input to the excitation is independent of the temporal order of activation. This feature combined with the observation that only few fibers converge on each neuron suggests that each fiber contributes equally to the generation of action potentials, ensuring high fidelity of information transfer.

### The nature and circuitry of the feed forward inhibition

Feed-forward inhibition is a common motive that serves to confine incoming excitation and provide well-defined time windows for synaptic integration (Swadlow, 2002; Priebe and Ferster, 2008). Our results compellingly demonstrate that a powerful, short-latency inhibition is activated at the first stage of afferent input integration. This finding is in agreement with the report that some interneurons in the DON show short latency responses (Duman, 1997). Thus, it is likely that the inhibition triggered by afferent fibers stimulation represents a feed-forward rather than feedback circuit. Moreover, the inhibitory input appears to be capable of complete elimination of the excitatory response evoked by the afferent nerve (Figure 8C) while having no effect on the parallel fiber input (Figure 8D), indicating that the neuronal compartment that house the parallel fiber input is electrically isolated from the compartment that receives the afferent input. Thus, we suggest that the inhibitory input driven by the afferent nerve represents a feed forward circuitry that at least partially is of pre-synaptic in origin (see also Paul and Roberts, 1977b).

Finally we found that at low stimulus intensity one can activate the excitatory input without activating the inhibitory pathway. Furthermore, the inhibitory effect, as a function of stimulus intensity, saturates before the excitation. Thus, it is likely that the fibers that evoke the excitation in a AEN differ from those that activate the inhibition. The saturation of the inhibition combined with the high variability in the response at intermediate intensities indicates that there are fewer afferent fibers that contribute to the inhibition than to the excitation.

## Concluding remarks

The DON serves as the first stage in which self-generated signals caused by the shark's own activity are predicted and cancelled. Recording from the afferent nerve shows that it carries both the predicted and unpredicted signals, while the parallel fiber input provides the AEN with information about the expected electrical field (Bodznick et al., 1999). It follows that either the excitatory input of the parallel fibers sums with the excitatory afferent input in a way that will bias the overall response toward the unexpected or that the inhibition triggered by the parallel fibers, via inhibitory interneurons, eliminates all signals related to expected sensations. The low threshold of AEN activation described here strongly argues against the first possibility. Furthermore, the possibility that the parallel fiber compartment is electrically isolated from the afferent compartment argues against any interactions between these two inputs at the level of the AEN. Our *in vitro* data suggests that a possible site for such interactions is a presumptive inhibitory interneuron that is activated by the afferent nerve. However, this possibility needs to be further examined in living animal.

### Conflict of interest statement

The authors declare that the research was conducted in the absence of any commercial or financial relationships that could be construed as a potential conflict of interest.

## References

[B1] Adams W. B. Parnas I. Levitan I. B. (1980). Mechanism of long-lasting synaptic inhibition in Aplysia neuron R15. J. Neurophysiol. 44, 1148–1160 6256509 10.1152/jn.1980.44.6.1148

[B2] Biesdorf S. Malinvaud D. Reichenberger I. Pfanzelt S. Straka H. (2008). Differential inhibitory control of semicircular canal nerve afferent-evoked inputs in second-order vestibular neurons by glycinergic and GABAergic circuits. J. Neurophysiol. 99, 1758–1769 10.1152/jn.01207.2007 18256163

[B3] Bodznick D. Montgomery J. C. Carey M. (1999). Adaptive mechanisms in the elasmobranch hindbrain. J. Exp. Biol. 202, 1357–1364 10210676 10.1242/jeb.202.10.1357

[B4] Bodznick D. Schmidt A. W. (1984). Somatotopy within the medullary electrosensory nucleus of the little skate, Raja erinacea. J. Comp. Neurol. 225, 581–590 10.1002/cne.902250408 6736290

[B5] Brown J. E. Muller K. J. Murray G. (1971). Reversal potential for an electrophysiological event generated by conductance changes: mathematical analysis. Science 174, 318 10.1126/science.174.4006.318 5119107

[B6] Clusin W. T. Bennett M. V. (1979). The oscillatory responses of skate electroreceptors to small voltage stimuli. J. Gen. Physiol. 73, 685–702 10.1085/jgp.73.6.685 479810 PMC2215210

[B7] Duman C. H. (1997). Short-latency interneurons in the dorsal nucleus of the little skate, Raja erinacea. Brain Res. 771, 80–88 10.1016/S0006-8993(97)00859-7 9383011

[B8] Fields R. D. Ellisman M. H. (1985). Synaptic morphology and differences in sensitivity. Science 228, 197–199 10.1126/science.3975637 3975637

[B9] Fishelson L. Baranes A. (1998). Distribution, morphology, and cytology of ampullae of Lorenzini in the Oman shark, *Iago omanensis* (Triakidae), from the Gulf of Aqaba, Red Sea. Anat. Rec. 251, 417–430 9713980 10.1002/(SICI)1097-0185(199808)251:4<417::AID-AR1>3.0.CO;2-P

[B10] Hentschel H. Nearing J. Harris H. W. Betka M. Baum M. Hebert S. C. (2003). Localization of Mg2+-sensing shark kidney calcium receptor SKCaR in kidney of spiny dogfish, Squalus acanthias. Am. J. Physiol. Renal Physiol. 285, F430–F439 10.1152/ajprenal.00081.2002 12759228

[B11] Oertel D. Young E. D. (2004). What's a cerebellar circuit doing in the auditory system? Trends Neurosci. 27, 104–110 10.1016/j.tins.2003.12.001 15102490

[B12] Ospeck M. (2012). Fast negative feedback enables mammalian auditory nerve fibers to encode a wide dynamic range of sound intensities. PLoS ONE 7:e32384 10.1371/journal.pone.0032384 22412868 PMC3297600

[B13] Paul D. H. Roberts B. L. (1977a). Studies on a primitive cerebellar cortex. I. The anatomy of the lateral-line lobes of the dogfish, Scyliorhinus canicula. Proc. R. Soc. Lond. B Biol. Sci. 195, 453–466 10.1098/rspb.1977.0020 15265

[B14] Paul D. H. Roberts B. L. (1977b). Studies on a primitive cerebellar cortex. III. The projection of the anterior lateral-line nerve to the lateral-line lobes of the dogfish brain. Proc. R. Soc. Lond. B Biol. Sci. 195, 479–496 10.1098/rspb.1977.0022 15267

[B15] Pothmann L. Wilkens L. A. Hofmann M. H. (2012). Two modes of information processing in the electrosensory system of the paddlefish (*Polyodon spathula*). J. Comp. Physiol. A Neuroethol. Sens. Neural Behav. Physiol. 198, 1–10 10.1007/s00359-011-0681-2 21960281

[B16] Priebe N. J. Ferster D. (2008). Inhibition, spike threshold, and stimulus selectivity in primary visual cortex. Neuron 57, 482–497 10.1016/j.neuron.2008.02.005 18304479

[B17] Rall W. Burke R. E. Smith T. G. Nelson P. G. Frank K. (1967). Dendritic location of synapses and possible mechanisms for the monosynaptic EPSP in motoneurons. J. Neurophysiol. 30, 1169–1193 4293410 10.1152/jn.1967.30.5.1169

[B18] Rotem N. Sestieri E. Cohen D. Paulin M. Meiri H. Yarom Y. (2007). The functional architecture of the shark's dorsal-octavolateral nucleus: an *in vitro* study. J. Exp. Biol. 210, 2730–2742 10.1242/jeb.001784 17644688

[B19] Sierra F. Comas V. Buno W. Macadar O. (2007). Voltage-gated potassium conductances in Gymnotus electrocytes(AB). Neuroscience 145, 453–463 10.1016/j.neuroscience.2006.12.002 17222982

[B20] Swadlow H. A. (2002). Thalamocortical control of feed-forward inhibition in awake somatosensory ‘barrel’ cortex. Philos. Trans. R. Soc. Lond. B. Biol. Sci. 357, 1717–1727 10.1098/rstb.2002.1156 12626006 PMC1693091

[B21] Tricas T. C. New J. G. (1998). Sensitivity and response dynamics of elasmobranch electrosensory primary afferent neurons to near threshold fields. J. Comp. Physiol. A 182, 89–101 10.1007/s003590050161 9447716

[B22] Weiger T. M. Colombatto S. Kainz V. Heidegger W. Grillo M. A. Hermann A. (2007). Potassium channel blockers quinidine and caesium halt cell proliferation in C6 glioma cells via a polyamine-dependent mechanism. Biochem. Soc. Trans. 35, 391–395 10.1042/BST0350391 17371284

[B23] Wen B. Wang G. I. Dean I. Delgutte B. (2009). Dynamic range adaptation to sound level statistics in the auditory nerve. J. Neurosci. 29, 13797–13808 10.1523/JNEUROSCI.5610-08.2009 19889991 PMC2774902

